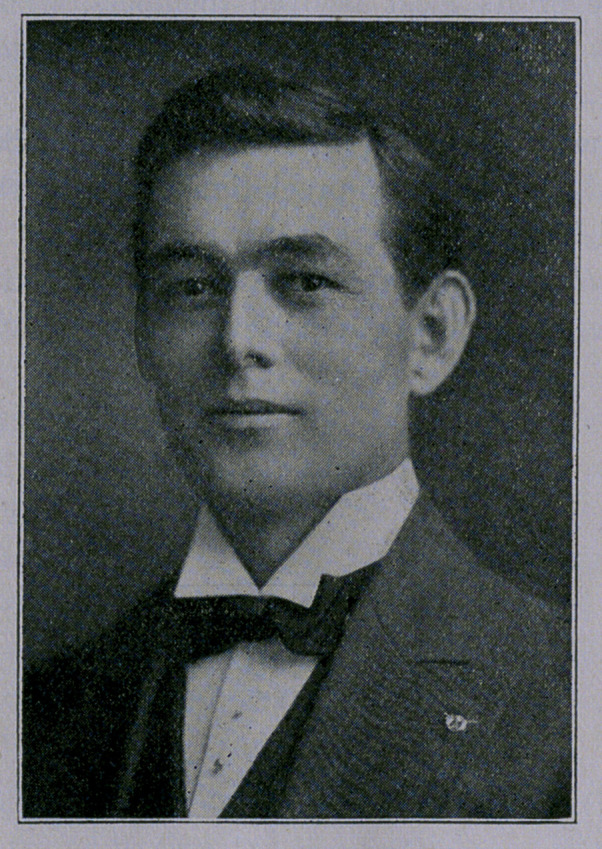# Hon. Jewel P. Lightfoot, Attorney General of Texas

**Published:** 1909-12

**Authors:** 


### HON. JEWEL P. LIGHTFOOT,

ATTORNEY GENERAL OF TEXAS.

Hon. R. V. Davidson, Attorney General, having resigned to enter the race for Governor, Governor Campbell promptly appointed Judge Lightfoot to succeed him. He will take up the discharge of his important duties January 1, proximo. For the several years of Governor Campbell's administration, Judge Lightfoot has been the senior Assistant Attorney General and has been charged with the management of the most important cases. To this brilliant young lawyer, accomplished scholar and chivalrous gentleman, a native Texan, the people are indebted for the great victory in the Waters-Pierce case, suit by the State of Texas for penalties and ouster, which was appealed to the Supreme Court at Washington. Judge Lightfoot achieved national renown in winning the case, and it was he who put the cash, nearly two million dollars, in the treasury vaults at Austin. It goes without saying that he will be a candidate for Attorney General at next election. I doubt if he will have any opposition. He is the most popular man in the State. He has always favored medical legislation, and it was he who gave the opinion that the physicians have a right to fix fees for services, and that in so doing they did not encroach on the anti-trust laws. This alone will make his candidacy of interest to doctors. He is at the head of the Woodmen's great organization, 135,000 strong in Texas, having been elected Chief Consul by a practically unanimous vote. He is a Shriner and a Knight Templar. Mr. Lightfoot was one of four persons in Texas invited to participate in the National Conference on Criminal Law and Criminology held at Chicago last June in commemoration of the fiftieth anniversary of the founding of the Northwest University School of Law, and to be the guest of the University. Last, he is a clean, high-minded, honorable man, to whose skirts there will never attach any suggestion of what we understand as "practical politics." THE JOURNAL predicts for him a great career of usefulness to his fellow man and the world. I expect to see him Governor some day. So mote it be.

## Figures and Tables

**Figure f1:**